# Economic costs of cigarette smoking among adolescents in Nigeria

**DOI:** 10.1007/s10389-021-01644-5

**Published:** 2021-10-27

**Authors:** Adesola O. Olumide, Amir Shmueli, Emmanuel S. Adebayo, Olayemi O. Omotade

**Affiliations:** 1 Institute of Child Health, College of Medicine, University of Ibadan and University College Hospital, Ibadan, Nigeria; 2 Department of Health Management and Economics, School of Public Health, Hebrew University of Jerusalem, Jerusalem, Israel

**Keywords:** Economic costs; cigarette smoking, Adolescents, Illness costs, Lung cancer

## Abstract

**Background:**

Cigarette smoking is an established cause of preventable death and often initiated during adolescence. We estimated the short- and long-term costs of cigarette smoking among currently smoking adolescents in Nigeria.

**Methods:**

A cross-sectional survey among adolescents in Oyo state, Nigeria and a review of mortality records of patients managed for lung cancer in a tertiary facility in Ibadan, Nigeria were conducted. Short-term costs estimated were: (a) average weekly costs of purchasing cigarettes by currently smoking adolescents, and (b) costs of managing at least an episode of chronic cough occurring within 12 months of the survey. Long-term costs were limited to: (a) life-time expenditure on purchasing cigarettes, and (b) direct medical and non-medical (transportation) costs of managing lung cancer. Long-term costs were first projected to the approximate year when the adolescents (mean age:16.0 ± 1.8 years) might be diagnosed with lung cancer based on the average age at presentation with symptoms of lung cancer obtained from the records (59.8 years), and then discounted to 2020 prices. This was estimated as 44 years from the base year (2020). Costs were reported in 2020 prices in Nigerian Naira (NGN) and US dollar (USD) equivalent using the Central Bank of Nigeria, June 2020 exchange rate of USD 1: NGN 360.50.

**Results:**

Approximately 3.8% of the adolescents were current cigarette smokers. Average weekly expenditure on cigarettes was NGN 306.82 ± 5.74 (USD 0.85 ± 0.02). About 26% had experienced at least an episode of chronic cough which cost them an average of NGN 1226.81 ± 6.18 (USD 3.40 ± 0.02) to manage. Total future costs of cigarette smoking in 2020 prices for the 43 adolescents who were current smokers in the event that they develop lung cancer were approximately NGN 175.7 million (USD 487.3 thousand), NGN 871.8 million (USD 2.4 million) and NGN 4.6 trillion (USD 12.7 million) at assumed annual inflation rates of 10%, 15%, and 20% respectively and discount rate of 4.25%.

**Conclusion:**

The estimated economic costs of smoking were very high. Efforts to prevent smoking initiation among adolescents in our study area should be intensified. Interventions to subsidize the medical cost of health-related consequences of cigarette smoking are also required, especially as treatment costs are currently largely borne out-of-pocket.

**Supplementary Information:**

The online version contains supplementary material available at 10.1007/s10389-021-01644-5.

## Background

Cigarette smoking is an important public health issue globally (Drope et al. 2018). Although smoking rates appear to have stalled globally, rates are increasing in many countries in sub-Saharan Africa and among adolescents (i.e., individuals aged 10 to 19 years) (Drope et al. 2018). The increasing rates of smoking among adolescents can be attributed to the fact that they are increasingly being targeted by tobacco companies, especially in low- and middle-income countries where systems for enforcing global and national tobacco control laws are fragile (Benjamin 2012; National Center for Chronic Disease Prevention and Health Promotion (US) Office on Smoking and Health 2012). In addition, adolescents are at a stage in life when they have a predisposition to experiment (McNeely et al. 2010). Many adolescents subsequently become established smokers after experimenting as they become addicted to tobacco. The prevalence of smoking among adolescents shows marked variation across and within countries. (Inchley and Currie 2013; Wang et al. 2019). Available data from Nigeria indicate that smoking prevalence also varies within the country. Results from Oyo state revealed that about 3.5% of students aged 13–15 years were current smokers (GYTS Nigeria 2008). Adebiyi et al. (2010), in a study of out-of-school adolescents, reported that 11.6% were current users of tobacco (cigarettes and snuff); and Olumide et al. (2014), in their study among adolescents aged 15–19 years residing in vulnerable communities in Ibadan, Nigeria, found that approximately 3.8% of the adolescents had smoked cigarettes within 30 days of the study.

Cigarette smoking has been linked to several short- and long-term health conditions affecting virtually all systems in the body. Some of these include oral and lung cancer, cancers of the gastrointestinal tract, urinary bladder cancer, and other effects on the cardiovascular and reproductive systems (Ezzati et al. 2002; Esson and Leeder 2004; US Department of Health and Human Services 2004; Cornfield et al. 2009; Pesch et al. 2012; Proctor 2012). In addition to the health implications of cigarette smoking, there are significant cost implications from managing smoking-related illnesses and loss of income from sickness, absenteeism, and premature death (Rice et al. 1991; French and Martin 1996; Szucs et al. 2001; Cawley and Ruhm 2011). These economic losses affect individuals, families, societies, and nations (Cawley and Ruhm 2011; WHO 2014;) and estimates of economic losses reveal the immense burden attributable to smoking.

Cost of illness studies estimate the burden of a disease or diseases to society in monetary terms (Rice 1994; Rice 2000). In order to facilitate comparability of findings across studies, guidelines for cost of illness studies that highlight key concepts that should be reported have been developed. Some of these include the study perspective, the approach for cost estimation, and the type of costs calculated. The study perspective could be societal, health care system, government, study participants and their families, or a third-party payer’s perspective (Hodgson 1994; Rice 2000). A prevalence or life-time (incidence) approach could be adopted, and costs could be direct, indirect, or intangible (Hodgson and Meiners 1982; Segel 2006). Direct costs are the expenses incurred because of the illness (Rice et al. 1985; Segel 2006). They can be further categorized as medical (Hodgson and Meiners 1982) or non-medical costs (Hodgson and Meiners 1982; Segel 2006). Indirect costs are, “the value of lost production because of reduced working time as a result of the illness under study" (WHO 2009), while intangible costs include “psychosocial” costs such as costs of pain and suffering (Hodgson and Meiners 1982; WHO 2009). In addition to providing the burden of disease in monetary terms, cost of illness studies are very useful for decision-making by policymakers (Segel 2006).

A review of existing literature revealed that although there are some cost studies originating from low- and middle-income countries (Hoang Anh et al. 2016; Boachie et al. 2021), the overwhelming majority are from high-income countries (Oster et al. 1984; Barendregt et al. 1997; Sung et al. 2006). Our literature search on costs of smoking in Nigeria revealed a report by Owoeye and Olaniyan (2015) describing the economic cost of tobacco-related diseases, and their findings support existing reports of the very high costs of smoking-attributable illnesses (Owoeye and Olaniyan 2015). Their estimates covered costs of long-term smoking-attributable diseases, but did not include expenditure on cigarettes or short-term smoking-related diseases. The aim of the current paper is to document the short-term and future costs of cigarette smoking among currently smoking adolescents in Oyo state, Nigeria. We focus on adolescents for a number of reasons. First, smoking initiation often occurs during this period and is likely to lead to tobacco addiction, resulting in a longer duration of lifetime use of cigarettes and subsequently considerable health effects (National Center for Chronic Disease Prevention and Health Promotion (US) Office on Smoking and Health 2012). Second, there is evidence of targeting of young people by tobacco companies in Nigeria through various strategies such as easy access to cigarettes and other tobacco products around schools, marketing of brands of cigarette (menthol and flavoured) that appeal to young people, sponsorship of concerts, and low pricing of tobacco products, amongst others (Isip and Calvert 2020; Nigeria Tobacco Control Research Group 2017). Finally, many of the existing measures for protecting adolescents from smoking initiation contained in the WHO Framework Convention on Tobacco Control (WHO FCTC) and Nigeria Tobacco Control Act, 2015 are inadequately implemented (Isip and Calvert 2020; WHO 2019). Our findings would thus provide information that could be useful for advocating for better enforcement and implementation of measures that prevent initiation of cigarette smoking among adolescents.

## Methods

Data for the current paper were obtained as part of a larger study on prevalence and costs of health-risk behaviours among adolescents. The study incorporated a questionnaire survey of in-and out-of-school adolescents, a review of health facility mortality records from a teaching hospital in Ibadan, Nigeria, costing of relevant health care, and other relevant non-medical commodities through expert enquiries. The questionnaire survey was conducted in urban (Ibadan North) and rural (Ibarapa Central) local government areas (LGAs) in Oyo state, Southwest Nigeria.

### Study participants

These comprised in-school and out-of-school adolescents (adolescents who had never been enrolled in school or who had been out of school for at least 1 year and had no immediate plans to continue with their education).

### Sampling technique

A multi-stage sampling technique was used to select in-school adolescents from senior classes in selected private and government-owned secondary schools. Out-of-school adolescents were selected using multi-stage sampling from locations where they routinely congregate such as market places, bus and taxi stops, etc. within both LGAs.^1^

### Scope of the study

We estimated the short and long-term costs of cigarette smoking among currently smoking adolescents. Short-term costs comprised:

(i) Average cost spent on purchasing cigarettes per week.
(ii) Average cost spent by the currently smoking adolescents to manage at least an episode of chronic cough (i.e., recurrent or persistent cough for which care was sought outside the home) experienced within 1 year prior to data collection.

Long-term costs were estimated using an incidence-based approach. The following costs were estimated:

(i) Life-time expenditure on purchasing cigarettes from the adolescent years until the approximate time the adolescent develops a smoking-related long-term illness, assuming the adolescent continues smoking at current rates, and
(ii) The average direct cost of terminal care for managing lung cancer. The causal association between cigarette smoking and conditions such as lung cancer and chronic cough (which could be a symptom of chronic obstructive pulmonary disease (COPD), has been established (US Department of Health and Human Services 2004) and this informed our choice of these two conditions. Estimation of the long term cost of illness was guided by the cost-of-illness and health-risk behaviours approach (Rice 1966; Chisholm et al. 2010). Healthcare costs were obtained using a bottom-up approach which involved multiplying the average cost of terminal care for lung cancer per person by the prevalence of the illness (Bloom et al. 2001). We calculated healthcare costs from the perspective of patients and their families (Hodgson 1994), and costs were limited to direct medical and direct non-medical (transportation) costs.

### Data collection

Trained research staff obtained information on smoking behaviour, costs of purchasing cigarettes, and cost of care for chronic cough from the selected adolescents with the aid of a semi-structured questionnaire. Costs were adjusted to their 2020 values using the average annual inflation rate of 12.89% reported by the Central Bank of Nigeria (CBN) (Central Bank of Nigeria (CBN) n.d.-a) between 2015 (when the data was obtained) and 2020.

Details of the procedures for determining the direct medical costs of terminal care for lung cancer (comprising clinic consultation, admission, investigations, drugs and other consumables, and service charges), and non-medical costs (transportation costs for return trips to the hospital for clinic appointments and admissions) have been described in another paper (Olumide et al. 2021). All costs were reported in 2020 prices. These prices were converted to their US dollar equivalent by applying the June 2020 exchange rate of USD 1: NGN 360.50 reported by the Central Bank of Nigeria (Central Bank of Nigeria (CBN) n.d.-b), to facilitate comparison with other studies.

### Data management

#### Study variables

### Cigarette smoking patterns among the adolescents

- Ever smoked cigarettes: history of ever smoking more than a few puffs of cigarette in the individual’s lifetime.
- Current cigarette smoking: smoked a cigarette at least once within 30 days of the start of the study.

### Cost of cigarette smoking among currently smoking adolescents in Ibadan

- Short-term costs of cigarette smoking:
  i. Average cost of purchasing cigarettes per week.
  ii. Average amount spent on treatment for at least one episode of chronic cough.
  Data on costs of purchasing cigarettes and costs of managing chronic cough were skewed. Costs were thus log transformed and the mean and standard deviation determined. The log transformed means and standard deviations were then back-transformed and values reported.

b. Long-term costs of cigarette smoking among the currently smoking adolescents
  This comprised (i) estimated lifetime expenditure on cigarettes and (ii) direct costs of terminal care for lung cancer. Long-term costs were projected to the approximate time when the adolescents might develop lung cancer if they continued smoking. The approximate year of a future diagnosis of lung cancer was determined by subtracting the average age of the adolescents in the current study (16.0 ± 1.8 years) from the average age at diagnosis of the patients with lung cancer (59.8 years) obtained from hospital mortality records. This gave an estimate of 44 years which was added to the study base year (2020). Thus, we assumed that the currently smoking adolescents might develop lung cancer in 2064.

i. Estimated cost of purchasing cigarettes from 2020 to 2064 discounted to 2020 prices assuming the adolescents continue smoking at the same rate from 2020 until demise from lung cancer in 2064 was calculated using Eq.(1) below:

1 $$ {Cost}_{cigarette\_2020-2064}={\displaystyle \begin{array}{c}{Cost}_{cigarette2020}+{Cost}_{cigarette2020}\ast {\left(1+ IR/1+ DR\right)}^1+{Cost}_{cigarette2020}\\ {}\ast {\left(1+ IR/1+ DR\right)}^2+{Cost}_{cigarette2020}\ast {\left(1+ IR/1+ DR\right)}^3+{Cost}_{cigarette2020}\ast \\ {}{\left(1+ IR/1+ DR\right)}^4\cdots \cdots \cdots \cdots +{Cost}_{cigarette2020}\ast {\left(1+ IR/1+ DR\right)}^{43}\end{array}} $$

Where

Cost_cigarette2020_: Average cost of purchasing cigarettes per person in 2020 prices
Cost_cigarette_2020–2064_: Cost of purchasing cigarettes between 2020 and 2064 discounted to 2020 prices
IR: Assumed annual inflation rate from 2020 to 2064

Three probable annual inflation rates (IR) (10%, 15%, and 20%) were used.

DR: Annual discount rate of 4.25% specified by the Central Bank of Nigeria (CIA 2017)

ii. Average cost of terminal care for lung cancer in the event that the adolescent develops lung cancer in the future as a consequence of cigarette smoking

The direct (medical and transportation) costs of terminal care for lung cancer estimated from the hospital records (Olumide et al. 2021) were projected to the approximate time when the adolescents could develop lung cancer if they continued smoking (44 years from the base year — 2020). (See Appendix Table A1 in Additional File 1).

Based on the assumption that the costs would inflate at the same rate (using assumed annual inflation rates of 10, 15, and 20%), the 2020 prices for direct cost of terminal care for lung cancer was first projected to the anticipated value in the year in which lung cancer might be detected (i.e., 2064). These costs were then discounted to their 2020 prices by applying an annual discount rate (DR) of 4.25% specified by the Central Bank of Nigeria (CIA 2017); (See Appendix Table A2 in Additional File 1). The direct cost of terminal care for lung cancer per person in 2064 discounted to 2020 prices was calculated thus (Eq. 2):

2 $$ \mathsf{Future}\ \mathsf{Cost}\ {\mathsf{of}\ \mathsf{care}}_{\mathrm{Lung}\ \mathrm{Ca}\_2064}=\left(\mathsf{Cost}\ {\mathsf{of}\ \mathsf{care}}_{\mathrm{Lung}\ \mathrm{Ca}\_2020}\right)\ \mathsf{x}\ {\left[\left(1+\mathrm{IR}\right)/\left(1+\mathrm{DR}\right)\right]}^{\mathrm{number}\ \mathrm{of}\ \mathrm{years}-1} $$

Where,

Future Cost of care_Lung Ca_2064_: Future cost of care for Lung Ca per person in 2064.
Cost of care_LungCa_2020_: Cost of terminal care for Lung CA in 2020.
IR: Assumed annual Inflation rate.
DR: Annual discount rate.

The estimated long-term cost of cigarette smoking per person in 2064 discounted to 2020 prices (Totalcost_cigarettesmoking_ /person in 2020 prices) was obtained by adding the lifetime costs of purchasing cigarettes and the future cost of terminal health care adjusted for the life-time probability of dying from lung cancer before 75 years of age for male and female current smokers (Peto et al. 2000) – Eq. (3).

3 $$ {\displaystyle \begin{array}{c}{\mathrm{Totalcost}}_{\mathrm{cigarette}\mathrm{smoking}}/\mathrm{person}\ \mathrm{in}\ 2020\ \mathrm{prices}={\mathrm{Cost}}_{\mathrm{cigarette}\_2020\hbox{--} 2064}+\\ {}\Big({\mathrm{LifetimePr}}_{\mathrm{lungca}}\ast \mathrm{Future}\ \mathrm{Cost}\ {\mathrm{of}\ \mathrm{care}}_{\mathrm{Lung}\ \mathrm{Ca}\_2064\Big)}\end{array}} $$

Where:

Totalcost_cigarettesmoking_ /person: Estimated cost of cigarette smoking per person in 2064 discounted to 2020 prices.
Cost_cigarette_2020–2064_: Cost of purchasing cigarettes between 2020 and 2064 discounted to 2020.
LifetimePr_lungca_: Life time probability of death from Lung Ca given that the adolescent smokes cigarette.
Future Cost of care_Lung Ca_2064_: Cost of care for lung cancer per person in 2064 discounted to 2020 values.

The lifetime risks of death from lung cancer (LTR_lungca_) by smoking status obtained from literature are shown in Table 1 below:

**Table 1:** Extract from data on cumulative lifetime risk of lung cancer among smokers (Peto et al. 2000)

Smoking status by gender		Cumulative life-time risk (%)
Current smoker	Male	15.9
	Female	9.5

The estimated cost of cigarette smoking for all the currently smoking adolescents in the survey in 2020 prices was estimated by multiplying the estimated total cost of cigarette smoking per person in 2064 discounted to 2020 prices (Totalcost_cigarettesmoking_ /person in 2020 prices) by the total number of currently smoking adolescents (Eq. 4 below):

4 $$ {\mathsf{Totalcost}}_{\mathsf{cigarettesmoking}}/\mathsf{person}\ \mathsf{in}\ \mathsf{2020}\ \mathsf{prices}\times \mathsf{43}\ \mathsf{currently}\ \mathsf{smoking}\ \mathsf{adolescents} $$

### Sensitivity analysis

This was conducted by applying the estimated total cost of cigarette smoking per person in 2064 discounted to 2020 prices to the prevalence of adolescent current cigarette smokers in: (i) Nigeria and (ii) Kano state — a state with a high prevalence of cigarette smoking in Nigeria (GYTS Nigeria 2008).

### Ethical statement

Ethical approval was obtained from the Oyo State Ethics Committee (AD13/479/193), Ministry of Health, Secretariat. Approval was also obtained from the management of the teaching hospital for review of patient records, and all identifiers were excluded from the data.

For adolescents aged < 16 years, informed consent was obtained from their parent/ caregiver and assent obtained from the adolescents. Informed consent was obtained from adolescents aged 16 years and above and those who were < 16 years who were considered to be mature minors.

## Results

### Socio-demographic characteristics of the adolescents

A total of 1169 adolescents were approached to participate in the quantitative survey, of whom 1142 agreed to participate, giving a response rate of 97.7%. The mean age of the respondents was 16 ± 1.8 years; 56.0% were male, and 84.0% were currently residing with at least one parent.

### Prevalence of cigarette smoking by the adolescents

Forty-nine adolescents (4.3%) had ever smoked a whole cigarette. The mean age at first smoking (a whole cigarette) was 15 ± 2.2 years. Median age at first smoking was 15 (range 10–19) years. Forty-three adolescents (3.8%) smoked cigarettes within 30 days of the study. On the days they smoked, the adolescents smoked a median of 2.5 (range: 1–10) sticks of cigarette per day. On the average, ten adolescents (27.8%) smoked one stick of cigarette per day, 21 (58.3%) smoked two to five sticks of cigarette per day, and five (13.9%) between six and ten sticks of cigarette per day (Table 2).

**Table 2:** Current cigarette smoking patterns among the adolescents

Variable	*N*	%
Smoked during the last 30 days (*n *= 1142)		
Yes	43	3.8
No	1099	96.2
Average number of sticks of cigarette smoked per day (n= 36)*		
1 stick	10	27.8
2 to 5	21	58.3
6 to 10	5	13.9

*sub-totals are different because of non-response

### Cost of cigarette smoking among the adolescents

 These costs fall into two categories:

- Short-term costs of cigarette smoking among currently smoking adolescents in Oyo state, Nigeria:
  Average costs of purchasing cigarettes per year
  Average cost spent managing an episode of chronic cough
- Long-term costs of cigarette smoking among the currently smoking adolescents in Oyo state, Nigeria:
  Estimated average cost of cigarettes purchased from 2020 to 2064 per adolescent
  Estimated average cost of managing lung cancer in the future per person in 2064

#### Average costs of purchasing cigarettes per year

Thirty-seven of the 43 current smokers provided information on the average amount of money spent on purchasing cigarettes per week. The mean amount spent on purchasing cigarettes per week in 2020 prices was NGN 306.82 ± 5.74 (USD 0.85 ± 0.02), and the median amount spent was NGN 275.03 (18.33–4934.89); USD 0.76 (0.05–13.69). The average amount spent per year was NGN 15, 954.64.

$$ \mathsf{Average}\ \mathsf{cost}\ \mathsf{of}\ \mathsf{purchasing}\ \mathsf{cigarettes}\ \mathrm{per}\ \mathsf{week}=\mathsf{NGN}\ \mathsf{306.82}\pm \mathsf{5.74}\ \left(\mathsf{USD}\ \mathsf{0.85}\pm \mathsf{0.02}\right)\mathrm{Annual}\ \mathrm{average}\ \mathrm{cost}\ \mathrm{of}\ \mathrm{purchasing}\ \mathrm{cigarettesper}\ \mathrm{person}\ \mathrm{in}\ 2020\ \left({\mathrm{Cost}}_{\mathrm{cigarette}2020}\right)=\mathrm{NGN}\ 306.82\times 52\ \mathrm{weeks}=\mathrm{NGN}\ 15,954.64 $$

#### Average cost spent managing an episode of chronic cough

Thirteen adolescents who had ever smoked reported that within the last 12 months, they had experienced at least an episode of recurrent or persistent cough. None of the adolescents reported history of chronic cough due to tuberculosis or previously diagnosed asthma. The mean costs spent to manage this cough was NGN 1226.81 ± 6.18 (US$ 3.40 ± 0.02) and median cost was NGN 1162.19 (NGN 183.35–9189.19) [US$ 3.22 (0.51–25.49)];.

#### Parameters for estimating long-term costs of cigarette smoking

- Average cost of purchasing cigarettes per adolescent per annum (2020 prices) = NGN 15,954.64
- Average cost of purchasing cigarettes per adolescent per annum (2020 prices) = USD 44.26
- Average cost for (49.2 days) of terminal care for lung CA per person (2020 prices) (Olumide et al. 2021) = NGN 510,152.62
- Average cost of terminal care for lung CA per person (2020 prices) USD = USD 1415.13
- Average inflation rate in Nigeria (2015 to 2020) = 12.89%
- Average assumed inflation rates 2020 to 2064 = 10%; 15% and 20%
- Average assumed discount rate = 4.25%
- Cumulative lifetime risks of developing lung cancer in current smokers (male) = 0.159
- Cumulative lifetime risk of developing lung cancer in current smokers (female) = 0.095
- Prevalence of cigarette smoking among study adolescents (all) = 3.8%
- Prevalence of cigarette smoking among study adolescents (male) = 5.9%
- Prevalence of cigarette smoking among study adolescents (female) = 1.0%
- Number of current male adolescent smokers = 38
- Number of current female adolescent smokers = 5
- Total number of current adolescent smokers = 43

#### Estimated average cost of cigarettes purchased from 2020 to 2064 per adolescent

The average costs of purchasing cigarettes from 2020 to 2064 discounted to 2020 prices were NGN 2,781,368.66 (USD 7715.31), NGN 11,457,604.94 (USD 31,782.54) and NGN 51,453,891.47 (USD 142,729.24) at assumed inflation rates of 10%, 15%, and 20% respectively.

Equation (1):

$$ {\mathsf{Cost}}_{\mathsf{cigarette}\_\mathsf{2020}\hbox{--} \mathsf{2064}}={\mathsf{Cost}}_{\mathsf{cigarette}\mathsf{2020}}+{\mathsf{Cost}}_{\mathsf{cigarette}\mathsf{2020}}\ast {\left(1+\mathrm{IR}/1+\mathrm{DR}\right)}^1+{\mathrm{Cost}}_{\mathrm{cigarette}2020}\ast {\left(1+\mathrm{IR}/1+\mathrm{DR}\right)}^2+{\mathrm{Cost}}_{\mathrm{cigarette}2020}\ast {\left(1+\mathrm{IR}/1+\mathrm{DR}\right)}^3+{\mathrm{Cost}}_{\mathrm{cigarette}2020}\ast {\left(1+\mathrm{IR}/1+\mathrm{DR}\right)}^4\dots \dots \dots \dots \dots +{\mathrm{Cost}}_{\mathrm{cigarette}2020}\ast {\left(1+\mathrm{IR}/1+\mathrm{DR}\right)}^{43} $$

- Average cost of purchasing cigarettes per week in 2020 prices = NGN 306.82 (5.74)
- Annual cost of purchasing cigarettes/ person in 2020 (Cost_cigarette2020_) = NGN 306.82 × 52 weeks = NGN 15,954.64.
- IR = Assumed annual inflation rate (10%, 15%, 20%)
- DR = Annual discount rate = 4.25%
- Cost_cigarette_2020–2064_ (1R =10%) = NGN 2,781,368.66 (USD 7715.31)
- Cost_cigarettel_2020–2064_ (1R =15%) = NGN11,457,604.94 (USD 31,782.54)
- Cost_cigarete_2020–2064_ (1R =20%) = NGN 51,453,891.47 (USD 142,729.24)

#### Estimated average cost of managing lung cancer in the future per person in 2064

The estimated average costs of terminal care for managing lung cancer per person in 2064 discounted to 2020 prices were NGN 5,132,333.23 (USD 14,236.71); NGN 34,708,553.92 (USD 96,278.93) and NGN 216,382,913.80 (USD 600,230.00) at assumed inflation rates of 10%, 15%, and 20% respectively. See Table 3 below.

**Table 3:** Cost of care for managing one person for lung cancer in 2064 (discounted to 2020 prices)

Cost of care for lung cancer for 49.2 days, 2020 cost of care_LungCa_2020_	Estimated inflation rates	Future cost of care_Lung Ca_2064_ in 2064 prices undiscounted	Future cost of care_Lung Ca_ in 2064 discounted to 2020 prices
NGN 510,152.62	10.0%	NGN 30,731,629.11	NGN 5,132,333.23
		USD 85,247.24	USD 14,236.71
	15.0%	NGN 207,829,530.50	NGN 34,708,553.92
		USD 57,6503.60	USD 96,278.93
	20.0%	NGN 1,295,667,906.00	NGN 216,382,913.80
		USD 3,594,086	USD 600,230.00

NGN = Nigerian Naira

**Table 4:** Total economic cost of smoking cigarettes per person with lung cancer in 2064, discounted to 2020 prices

Assumed annual inflation rates	Total inflated and discounted cost of purchasing cigarettes for 44 years	Estimated tax based on 29.73% of retail price of cigarettes	*Lifetime probability of developing lung cancer given that the adolescent smokes cigarette	Inflated and discounted costs of terminal care for lung cancer per person)	Total economic cost of smoking cigarettes per person with lung cancer in 2020 prices
(IR)	(Cost_cigarettes_2020–2064_)		(LifetimePr_Lung Ca_)	(Discounted cost of care in 2020 prices_Lung Ca_)	(Totalcost_cigarette_/person)
A	B	B.1	C	D	E = B + (C*D)
10%	NGN 2,781,368.66	826,900.90	(0.159+ 0.095)	NGN 5,132,333.23	NGN 4,084,981.3 (USD11331.43)
15%	NGN 11,457,604.94	3,406,345.95	(0.159+ 0.095)	NGN 34,708,553.92	NGN 20,273,577.64 USD 56,237.39
20%	NGN 51,453,891.47	15,297,241.93	(0.159+ 0.095)	NGN 216,382,913.80	NGN 106,415,151.6 USD 295,187.66

NGN = Nigerian Naira

Equation (2)

$$ \mathrm{Future}\ \mathrm{Cost}\ {\mathrm{of}\ \mathrm{care}}_{\mathrm{Lung}\ \mathrm{Ca}\_\mathsf{2064}}=\left(\mathsf{Cost}\ {\mathsf{of}\ \mathsf{care}}_{\mathsf{LungCa}\_\mathsf{2020}}\right)\ \mathsf{x}\ {\left[\left(\mathsf{1}+\mathsf{IR}\right)/\left(\mathsf{1}+\mathsf{DR}\right)\right]}^{\mathsf{number}\ \mathsf{of}\ \mathsf{years}-\mathsf{1}}\mathrm{Future}\ \mathrm{Cost}\ {\mathrm{of}\ \mathrm{care}}_{\mathrm{Lung}\ \mathrm{Ca}\_2064}=\left(\mathrm{Cost}\ {\mathrm{of}\ \mathrm{care}}_{\mathrm{Lung}\mathrm{Ca}\_2020}\right)\ \mathrm{x}\ {\left[\left(1+\mathrm{IR}\right)/\left(1+\mathrm{DR}\right)\right]}^{44\hbox{--} 1}\mathrm{Cost}\ {\mathrm{of}\ \mathrm{care}}_{\mathrm{Lung}\mathrm{Ca}\_2020}=\mathrm{N}\ 510,152.62\ \mathrm{for}\ 49.2\ \mathrm{days}\ \mathrm{on}\ \mathrm{terminal}\ \mathrm{care} $$

(Olumide et al. 2021)

Thus,

$$ \mathrm{Future}\ \mathrm{Cost}\ {\mathrm{of}\ \mathrm{care}}_{\mathrm{Lung}\ \mathrm{Ca}\_2064}=\left(\mathrm{510,152.62}\right)\ \mathrm{X}\ {\left(1.10/1.0425\right)}^{43} $$

The estimated total cost of cigarette smoking per person in 2064 discounted to 2020 prices (Totalcost_cigarettesmoking_ /person) was obtained using Eq. (3):

Equation 3

$$ {\mathsf{Totalcost}}_{\mathsf{cigarettesmoking}}/\mathsf{person}\ \mathsf{in}\ \mathsf{2064}\ \mathsf{in}\ \mathsf{2020}\ \mathsf{prices}={\mathrm{Cost}}_{\mathrm{cigarette}\_2020\hbox{--} 2064}+\left({\mathrm{LifetimePr}}_{\mathrm{lungca}}\ast \mathrm{Future}\ \mathrm{Cost}\ {\mathrm{of}\ \mathrm{care}}_{\mathrm{Lung}\ \mathrm{Ca}\_2064}\right) $$

The expected total lifetime cost of cigarette smoking for all the adolescents in the survey who smoke cigarettes in 2020 prices was estimated by multiplying the estimated total cost of cigarette smoking per person in 2064 discounted to 2020 prices (Totalcost_cigarettesmoking_ /person in 2020 prices) by the total number of currently smoking adolescents:

$$ {\mathsf{Totalcost}}_{\mathsf{cigarettesmoking}}/\mathsf{person}\ast \mathsf{Total}\ \mathsf{number}\ \mathsf{of}\ \mathsf{adolescents}\ \mathrm{who}\ \mathsf{smoke}\ \mathsf{cigarettes} $$

The estimated annual cost of smoking cigarettes in the event that the individual goes on to develop lung cancer was projected to the population of adolescents in the current study based on the prevalence of current cigarette smoking among the study population. 

#### Total economic cost of smoking cigarettes in 2020 prices for all adolescent current smokers

The total economic costs of smoking cigarettes includes the annual costs of purchasing cigarettes and the economic cost of care.

$$ \mathsf{Prevalence}\ \mathsf{of}\ \mathsf{current}\ \mathsf{cigarette}\ \mathsf{smoking}\ \mathsf{in}\ \mathsf{the}\ \mathsf{sample}=\mathsf{3.8}\%\mathrm{Total}\ \mathrm{number}\ \mathrm{of}\ \mathrm{adolescents}\ \mathrm{who}\ \mathrm{currently}\ \mathrm{smoke}\ \mathrm{cigarette}=43 $$

Total economic costs of smoking cigarettes discounted to 2020 prices for all 43 adolescents in the current study who smoke cigarettes in the event that they go on to develop lung cancer were estimated to be approximately NGN 175.7 million (USD 487,251.58), NGN 871,763,838.30 (USD 2,418,207.60) and NGN 4.58 trillion (USS 12.69 million) at annual inflation rates of 10%, 15%,and 20% respectively (Table 5).

#### Sensitivity analysis

Sensitivity analysis was conducted by applying different prevalence rates to the costs of smoking cigarettes. Two prevalence rates were considered: (1) the national prevalence rate of smoking among adolescents 15 to 19 years in Nigeria, using the prevalence reported by the GYTS data and applying this to the projected 2020 population of adolescents in Nigeria and (2) the smoking prevalence rates obtained in a state in Nigeria with the highest adolescent smoking prevalence rates obtained during the GYTS (Kano state), (GYTS Nigeria 2008).

##### Parameters for sensitivity analysis

Prevalence of cigarette smoking among adolescents (15 - 19 years) in Nigeria  = 3.8%.

Prevalence of cigarette smoking among adolescents (15 - 19 years) in Kano — high prevalence state  = 6.2%.

#### Projection of economic cost of cigarette smoking to national data

- 2006 Nigerian population^2^ = 140,431,790
- Projected national population in 2020 (Growth rate — 3.2%) = 218,263,540
- Projected opulation of adolescents 15–19 years in 2020 (10.6% of the total population) = 3,135,935
- Prevalence of current cigarette smoking among adolescents aged 15–19 years in Nigeria^3^ = 3.8% (0.038)
- Estimated number of currently smoking adolescents = 879,166 (15–19 years)
- Population in Kano in 2006 = 9,401,288
- Population in 2020 = 14,811,255
- Projected population of adolescents 15–19 years in 2020 in Kano (10.4% of the total population) = 1,540,371
- Prevalence of current smoking in Kano = 6.2%
- Estimated number of currently smoking adolescents in Kano = 95,503

Total economic cost of current cigarette smoking in 2020 prices for adolescent cigarette smokers nationally and in Kano state in the event that the adolescents develop lung cancer are reported in Table 5. The estimated total economic costs of smoking for all adolescent current smokers in 2020 prices was in excess of USD 259 trillion nationally, and USD 28 trillion in Kano state, where the prevalence is about twice the national prevalence (Table 6)

**Table 5:** Total economic cost of consuming cigarettes in 2020 prices for all adolescents in the current study who smoke cigarettes in the event that they go on to develop lung cancer

Assumed annual inflation rates	Total economic cost of smoking cigarettes per person in 2020 prices	Total economic cost of smoking cigarettes in 2020 prices for all adolescents in the current study who smoke cigarettes
A	B	C = B x no. of adolescents currently smoking cigarettes (43)
IR	Totalcost_cigarettesmoking_ /person	
10%	NGN 4,084,981.30 USD11,331.43	NGN 175,654,195.90 USD 487,251.58
15%	NGN 20,273,577.64 USD 56,237.39	NGN 871,763,838.30 USD 2,418,207.60
20%	NGN 106,415,151.60 USD 295,187.66	NGN 4,575,851,518 USD 12,693,069.40

NGN = Nigerian Naira

**Table 6:** Total economic cost of current cigarette smoking in 2020 prices for adolescent cigarette smokers nationally and in Kano state in the event that the adolescents develop lung cancer

Assumed annual inflation rates	Total economic cost of cigarette smoking per person in 2020 prices	Total economic cost of current cigarette smoking in 2020 prices for adolescent cigarette smokers nationally and in Kano state	
		National prevalence 3.8%	Prevalence in Kano state, Nigeria 6.2%
		*Estimated n* smoking = 879,166	*Estimated n* smoking = 95,503
IR	Totalcost_cigarettesmoking_ /person		
10%	NGN 4,084,981.30 USD11,331.43	NGN 3,591,376,669,595.80 USD 9,962,207,987.38	NGN 390,127,969,093.90 USD 1,082,185,559.29
15%	NGN 20,273,577.64 USD 56,237.39	NGN 17,823,840,159,448.20 USD 49,442,001,216.74	NGN 1,936,187,485,352.92 USD 5,370,839,457.17
20%	NGN 106,415,151.60 USD 295,187.66	NGN 93,556,583,171,565.60 USD 259,518,954,643.23	NGN 10,162,966,223,254.80 USD 28,191,307,131.18

NGN = Nigerian Naira

.

## Discussion

This paper presents estimates of the current and future economic costs of cigarette smoking among adolescents in Nigeria. To the best of our knowledge this is the first paper presenting these costs estimates from Nigeria, as available literature is largely from developed countries. The prevalence of cigarette smoking among adolescents varies within and across countries (WHO 2019) (GYTS). Among our sample, 3.8% of adolescents were current smokers, i.e., they had smoked within 30 days of the study. This is comparable to findings from other studies conducted in our study area — Ibadan (GYTS Nigeria 2008; Olumide et al. 2014), but lower than findings from other cities in Nigeria. For instance, Salawu et al. (2010) found that about a third of adolescents in North Eastern Nigeria were current smokers (i.e., individuals who had smoked more than 100 cigarettes in their lifetime and were still smoking at the time of the survey). This difference in prevalence rates could have been due to differences in definition of current smokers used — we used a definition of adolescents who smoked in the last 30 days. Another factor which might have contributed to the lower rates found in our study was the difference in sampling strategy. Our adolescents were selected from within secondary schools and from out-of-school locations, whereas Salawu et al. (2010) selected all their participants from the market area. This could have resulted in an oversampling of adolescents who were out-of-school and/or no longer under parental supervision. These categories of adolescents are likely to have more freedom to engage in risky behaviours such as cigarette smoking and use of other psychoactive substances. Previous studies have also reported variation in the prevalence of cigarette smoking among adolescents in Nigeria (GYTS Nigeria 2008; Oyewole et al. 2018).

The mean cost of purchasing cigarettes per week spent by the adolescents was approximately NGN 306.82 ± 5.74 (USD 0.85 ± 0.02) in 2020 prices, although one adolescent claimed to spend as much as NGN 4934.89 (USD 13.69) on cigarettes every week. Akanonu et al. (2019) reported that the average costs of a pack of 20 cigarettes in a 2016 survey in Nigeria ranged from NGN 100 to about NGN 250, with an average of approximately NGN 190.00 (Akanonu et al. 2019). The WHO estimates of the cost of a 20-cigarette pack of the most-sold brand, adjusted for purchasing power of national currencies in 2018, in Nigeria was NGN 220.00 (equivalent to USD 0.72 at official exchange rates) (WHO 2019). Although the expenditure on cigarettes per week among our adolescents appear minimal, our findings reveal that the costs of cigarettes are low and readily affordable by adolescents. This makes it easy for adolescents to purchase and experiment with cigarette smoking and subsequently become established smokers. Keeping cigarette prices low is a recognized strategy for targeting young people used by the tobacco industry (Isip and Calvert 2020; Nigeria Tobacco Control Research Group 2017). To counter this strategy, WHO advocates a minimum of 70% of the retail price as excise tax on cigarettes, based on evidence that higher prices reduce tobacco consumption, especially among young people (Drope et al. 2018). In 2018, the Government of Nigeria approved a new tax policy on tobacco products which raised overall tax on tobacco control products to 29.73% (WHO 2019). This value is much lower than the minimum percentage stipulated by WHO and thus needs an upward review in order to achieve the desired reduction in demand for cigarettes. In addition to the short-term costs associated with purchasing cigarettes, we found that 13 (26.5%) of the 49 adolescents who had ever smoked experienced at least one episode of chronic cough in the 12-month period preceding the study. Approximately NGN 1008.38 (USD 3.31) and as much as NGN 6350.00 (USD 20.84) was spent on treatment. These costs represent money which could have been spent on other commodities, and the implications of this in a country where about 40% of the population have been classified as poor (National Bureau of Statistics, 2020) and health care costs are mostly borne out-of-pocket further highlight the need for government to intensify efforts to control cigarette smoking.

Similar to findings from other studies, our estimated long-term costs of cigarette smoking were very high. The total cost of cigarette smoking (i.e., the cost of purchasing cigarettes and the cost of medical care) per person who might develop lung cancer in 2064 (in 2020 prices) was approximately 106.4 million naira (295.2 thousand US dollars) at an assumed inflation rate of 20%. Corresponding costs for all the 43 current adolescent smokers was more than 4.6 trillion naira (about 12.7 million US dollars) and more than 259 trillion US dollars (in 2020 prices) when extrapolated to national adolescent smoking prevalence rates. Other cost-of-smoking studies have reported very high costs of smoking. For example, Oster et al. (1984) documented that in 1980, the healthcare costs for a 40-year-old man ranged from $20,000 for a person who smokes less than one pack of cigarettes per day to more than $56,000 for a person who smokes more than two packs of cigarettes per day. Hoang Anh et al. (2014) estimated that the total economic cost of smoking based on five smoking-related diseases in Vietnam was approximately US$1173.2 million in 2011, or 0.97% of the 2011 gross domestic product (Hoang Anh et al. 2016). Although the estimated costs associated with cigarette smoking vary significantly across studies for various reasons including differences in the range and sophistication of treatments, differences in healthcare costs, and differences in the duration of treatment within countries, it is evident that costs of cigarette smoking are astronomical, and efforts to prevent initiation of smoking by adolescents must be intensified.

## Conclusion

Our findings on the immediate and long-term costs of cigarette smoking among adolescents in Nigeria confirm the immense economic impact of cigarette smoking. The bulk of the costs would be incurred at a time when the adolescent should still be contributing meaningfully to national development. Whatever financial benefits are hoped to be made from cigarette sales can not be compared to the astronomical short- and long-term economic costs associated with cigarette smoking among adolescents who represent the future of a nation. These issues call for increased and urgent investments in interventions to prevent smoking initiation among adolescents. It should also be noted that these costs are conservative and do not take into consideration the indirect costs from inability to work or death which if included would give much higher estimates. We therefore recommend that government needs to be more pro-active in implementing the provisions of the Framework Convention on Tobacco Control (FCTC) and the Nigeria Tobacco Control Act so as to minimize initiation of cigarette smoking among adolescents in our setting. These measures include: (i) increasing taxes on cigarette in line with the WHO recommendations in order to ensure this increment achieves a substantial reduction in demand for cigarettes and (ii) enforcement of the ban on selling cigarettes in sticks and sale to minors to reduce access of cigarettes to adolescents. Our study has a few limitations which should be borne in mind when interpreting our findings.

1. The long-term healthcare costs were based on estimates of costs obtained for approximately 49 days of terminal care and do not include costs that would have been incurred for patients diagnosed in the early stages of lung cancer. Thus our estimates would be lower than actual costs. Our findings should thus be interpreted with this in mind. In our setting, many patients present in the late stages of the disease, making it difficult to capture all costs.
2. Cost estimates were based on direct medical and non-medical (transportation) costs and did not include indirect costs or intangible costs. Furthermore, we relied on participants’ report of short-term healthcare costs and expenditure on smoking. In order to promote accurate reporting of costs, participants were informed of the importance of reporting correct estimates, and we limited short-term health care costs to costs expended within the last 12 months and expenditure on cigarettes to amount spent per week.
3. The study was conducted among 1142 adolescents from a city in Southwest Nigeria. The results may therefore not be nationally representative of adolescents in Nigeria.

In spite of these limitations, we have presented reasonable estimates of the short- and long-term costs from cigarette smoking among adolescents in Nigeria, and this fills a major gap in the dearth of data on economic costs of smoking in Nigeria.

## Supplementary information

ESM 1 (DOCX 13 kb)

## Data Availability

The datasets used and/or analysed during the current study are available from the corresponding author on reasonable request.

## Footnotes

1 A list of locations where out-of-school adolescents are known to congregate was developed after informal discussions with adults who work with adolescents and a walk-though of the LGAs. Some of these locations were selected by random sampling and consenting respondents in the locations interviewed

2 Federal Republic of Nigeria Official Gazette. Abuja 2 February 2009. Legal Notice on Publication of 2006 Census Final Results. Pages B1–42. The Federal Government Printer, Abuja, Nigeria FGP16/22009/10,000 (OL 02)

3 GYTS Nigeria (2008) Global Youth Tobacco Survey for Nigeria. Abuja, Nigeria

## Abbreviations

CBN: Central Bank of Nigeria
DR: Annual Discount Rate
GYTS: Global Youth Tobacco Survey
HBSC: Health Behaviour in School-aged Children
IR: Inflation Rate
NGN: Nigerian Naira
UCH: University College Hospital
USD: United States Dollars
WHO: World Health Organization
