# Supplementary material for: Economic costs of cigarette smoking among adolescents in Nigeria
Source: Z Gesundh Wiss. 2021 Oct 27;30(7):1701–12. doi: 10.1007/s10389-021-01644-5 (PMC9246810; doi:10.1007/s10389-021-01644-5)
Supplement: Supplementary file 1 — (DOCX 13 kb) [file 10389_2021_1644_MOESM1_ESM.docx]

**Economic costs of cigarette smoking among adolescents in Nigeria**

Appendix 1

Table A1: Estimated time of diagnosis of the selected long-term illness attributable to cigarette smoking

| Risk behavior | Long-term illness related to the health-risk behavior | Average age at diagnosis for patients with the selected long-term illness | | Estimated number of years within which adolescent might develop the disease  (average age at diagnosis – average age of adolescents) | Approximate year of possible diagnosis for adolescents engaging in the risk behaviours |
| --- | --- | --- | --- | --- | --- |
| Cigarette smoking | Lung cancer | 59.8 | Source  Mortality records of the UCH ^a^ | 44 | 2064 |

a = University College Hospital (UCH) Ibadan, Mortality Records (unpublished)

Appendix 2

Table A2: Summary of variables for estimating future costs of Lung Ca

| Health-risk behavior | Long-term illness | Average time to diagnosis of illness (years) | Estimated Annual inflation rate | Cost of care_LungCa_2020_ | Future cost of care_2064 | Annual discount rate | Discounted value of Future cost of care in 2064 in 2020 prices |
| --- | --- | --- | --- | --- | --- | --- | --- |
| Cigarette smoking | Lung cancer | 44 | 10% | Cost of care_Lung ca_2020_ | Cost of care_Lung ca_2020_*1.10^43^ | 4.25% | Future cost of care_2064/1.0425^43^ |
|  |  |  | 15% | Cost of careL_ung ca_2020_ | Cost of care_Lung ca_2020_*1.15^43^ | 4.25% | Future cost of care_2064/1.0425^43^ |
|  |  |  | 20% | Cost of careL_ung ca_2020_ | Cost of care_Lung ca_2020_*1.20^43^ | 4.25% | Future cost of care_2064/1.0425^43^ |
